# Interleukin-6 Modulates Colonic Transepithelial Ion Transport in the Stress-Sensitive Wistar Kyoto Rat

**DOI:** 10.3389/fphar.2012.00190

**Published:** 2012-11-07

**Authors:** Dervla O’Malley, Timothy G. Dinan, John F. Cryan

**Affiliations:** ^1^Alimentary Pharmabiotic Centre, University College CorkCork, Ireland; ^2^Department of Physiology, University College CorkCork, Ireland; ^3^Department of Psychiatry, University College CorkCork, Ireland; ^4^Department of Anatomy and Neuroscience, University College CorkCork, Ireland

**Keywords:** capsaicin, veratridine, interleukin-6, irritable bowel syndrome, Wistar Kyoto, ussing chambers, electrophysiology

## Abstract

Immunological challenge stimulates secretion of the pro-inflammatory cytokine interleukin (IL)-6, resulting in variety of biological responses. In the gastrointestinal tract, IL-6 modulates the excitability of submucosal neurons and stimulates secretion into the colonic lumen. When considered in the context of the functional bowel disorder, irritable bowel syndrome (IBS), where plasma levels of IL-6 are elevated, this may reflect an important molecular mechanism contributing to symptom flares, particularly in the diarrhea-predominant phenotype. In these studies, colonic ion transport, an indicator of absorption and secretion, was assessed in the stress-sensitive Wistar Kyoto (WKY) rat model of IBS. Mucosa-submucosal colonic preparations from WKY and control Sprague Dawley (SD) rats were mounted in Ussing chambers and the basal short circuit current (*I*_SC_) was electrophysiologically recorded and compared between the strains. Exposure to IL-6 (1 nM) stimulated a secretory current of greater amplitude in WKY as compared to SD samples. Furthermore, the observed IL-6-mediated potentiation of secretory currents evoked by veratridine and capsaicin in SD rats was blunted in WKY rats. Exposure to IL-6 also stimulated an increase in transepithelial resistance in both SD and WKY colonic tissue. These studies demonstrate that the neuroexcitatory effects of IL-6 on submucosal plexi have functional consequences with alterations in both colonic secretory activity and permeability. The IL-6-induced increase in colonic secretory activity appears to neurally mediated. Thus, local increases in IL-6 levels and subsequent activation of enteric neurons may underlie alterations in absorpto-secretory function in the WKY model of IBS.

## Introduction

The functional gastrointestinal (GI) disorder, irritable bowel syndrome (IBS) is characterized by episodic bouts of abdominal pain, bloating, and altered bowel habit including diarrhea, constipation, or both. Although the pathophysiological changes underlying IBS are still being investigated, stress has been attributed a role in the initiation, exacerbation, and persistence of IBS symptom flares (Lydiard et al., 1993; Spiller, 2004; Fitzgerald et al., 2008). Additionally, a growing body of data implicates local activation of gut immune factors in the development and persistence of IBS symptoms (Quigley, 2006; O’Malley et al., 2011c). Mucosal biopsies from IBS patients express higher levels of T-cells, lymphocytes, and mast cells (Chadwick et al., 2002) and plasma samples from IBS patients exhibit altered pro-inflammatory cytokine profiles (Macsharry et al., 2008). Indeed, interleukin (IL)-6 has reproducibly been found to be elevated in plasma samples from IBS patients (Dinan et al., 2006, 2008; Liebregts et al., 2007; Clarke et al., 2009; Scully et al., 2010; McKernan et al., 2011).

As yet, the mechanisms that link altered cytokine profiles with the development of functional GI disorders such as IBS are poorly understood. However, there is growing evidence that IBS patients have altered GI permeability (Camilleri et al., 2012) and most pro-inflammatory cytokines have the capacity to influence intestinal epithelial permeability (Al-Sadi et al., 2009). Indeed, the importance of cytokines in neuromuscular dysfunction in the inflamed intestine has been demonstrated (Hurst et al., 1993; Ruhl et al., 1994), thus, with particular relevance to post-infective IBS, immunomodulation of enteric neurons by cytokines released from within the GI milieu may be important in the persistence of IBS symptomatology (Ruhl et al., 2001).

Increased IL-6 synthesis following administration of a cholinesterase inhibitor has been correlated with increased abdominal pain and bloating (Dinan et al., 2008) and IL-6 can modulate mucosal ion transport and epithelial permeability, in addition to enhancing cholinergically mediated neurotransmission in rodents (Natale et al., 2003). Moreover, both IL-1β and IL-6 act as excitatory neuromodulators in a subset of myenteric neurons via presynaptic inhibition of acetylcholine release (Kelles et al., 2000). IL-6 has also been shown to suppress nicotinic and noradrenergic neurotransmission in guinea-pig submucosal neurons (Xia et al., 1999). Previous studies from our group have shown expression of IL-6 receptors on a subset of rat colonic submucosal neurons. Exposure of these neurons to recombinant IL-6 results in increases in intracellular calcium [(Ca^2+^)_i_] levels, which in turn results in increased colonic secretion (O’Malley et al., 2011b).

The Wistar Kyoto (WKY) rat has been validated (Greenwood-Van Meerveld et al., 2005; Gibney et al., 2010; O’Malley et al., 2010a) as an appropriate pre-clinical model of IBS, displaying increased visceral sensitivity to colorectal distension and enhanced colonic motility and fecal output following exposure to a psychological stressor (Gibney et al., 2010; O’Malley et al., 2010a). Colonic morphology and goblet cell expression is also altered in this rat (O’Malley et al., 2010a) and it exhibits evidence of altered cytokine expression. Although plasma levels of IL-6 are not different between WKY and SD rats (unpublished observation), mucosal scrapings from WKY colons contain higher levels of IL-6 and excised WKY colons secrete more IL-6 than control Sprague Dawley (SD) colons. Moreover, these secretions stimulate calcium responses of greater amplitude in naïve submucosal neurons than the SD secretions (O’Malley et al., 2011a). These observations are comparable to studies carried out in the maternal separation (MS) model of IBS where MS secretions stimulated a larger response in submucosal neurons than control non-separated colonic secretions. Moreover, recombinant IL-6 was shown to stimulate an increase in secretory activity (O’Malley et al., 2011b).

Evidence is mounting that IL-6 has neuromodulatory effects that contribute to altered GI function, however it is currently unclear whether these effects translate into functional changes. The current studies use Ussing chamber electrophysiology to investigate absorpto-secretory function in WKY rats following exposure to IL-6 and compare these effects to the SD control strain, which has normal GI function.

## Materials and Methods

### Animals

Sprague Dawley and WKY rats (200–250 g) purchased from Harlan, UK were group-housed 4–6/cage and maintained on a 12/12 h dark-light cycle (08.00–20.00). All experiments were in full accordance with the European Community Council Directive (86/609/EEC) and the local University College Cork animal ethical committee.

### Tissue preparation

Distal segments of colon were excised from naïve SD or WKY rats and maintained in ice-cold bubbled (95% O_2_/5% CO_2_) Krebs saline consisting of (in mmol/L) NaCl, 117; KCl, 4.8; CaCl_2_, 2.5; MgCl_2_, 1.2; NaHCO_3_, 25; NaH_2_PO_4_, 1.2; and d-glucose, 11. Longitudinal and circular muscle layers were removed to prepare a mucosa-submucosal preparation for Ussing chamber electrophysiology as previously described (O’Malley et al., 2011b).

### Ussing chamber electrophysiology

Mucosa-submucosal preparations of distal colon were mounted in Ussing chambers (exposed area of 0.12 cm^2^) with 5 ml of Krebs solution (95% O_2_/5% CO_2_, 37^°^C) in the basolateral and luminal reservoirs. Tissues were voltage-clamped at 0 mV using an automatic voltage clamp (EVC 4000, World Precision Instruments, Sarasota, FL, USA) and the short circuit current (*I*_SC_) required to maintain the 0 mV potential was monitored as a recording of the net active ion transport across the epithelium. Experiments were carried out simultaneously in all chambers and connected to a PC equipped with DataTrax II software (WPI). This software was used to measure the peak response and resistance was calculated using Ohms law.

Based on previous evaluations of the pro-secretory effects of IL-6 in SD tissues (O’Malley et al., 2011b), it was determined that the peak response to IL-6 occurred within 10 min of application. Thus, this time point was used to compare the effects of IL-6 on secretion in WKY versus SD rats. Following a period of stabilization (30–60 min) and prior to addition of any reagents, transepithelial resistance (TER) was measured. Another measurement of TER was taken at the end of the experiment (60–90 min later) and the difference (Δ resistance) between the two measurements was calculated.

### Statistics

The data are represented as mean values ± the standard error of the mean (SEM). Students’ *t*-test and one-way ANOVA with Neumann Keuls *post hoc* test were used to compare groups. Two-way ANOVA was used to analyze strain and treatment effects as independent variables. *p* ≤ 0.05 was considered significant. All experiments were conducted in tissue taken from at least six different animals.

## Results

### *IL-6* evokes increased colonic secretion and transepithelial resistance in SD and *WKY* rats

**Figure 1:**
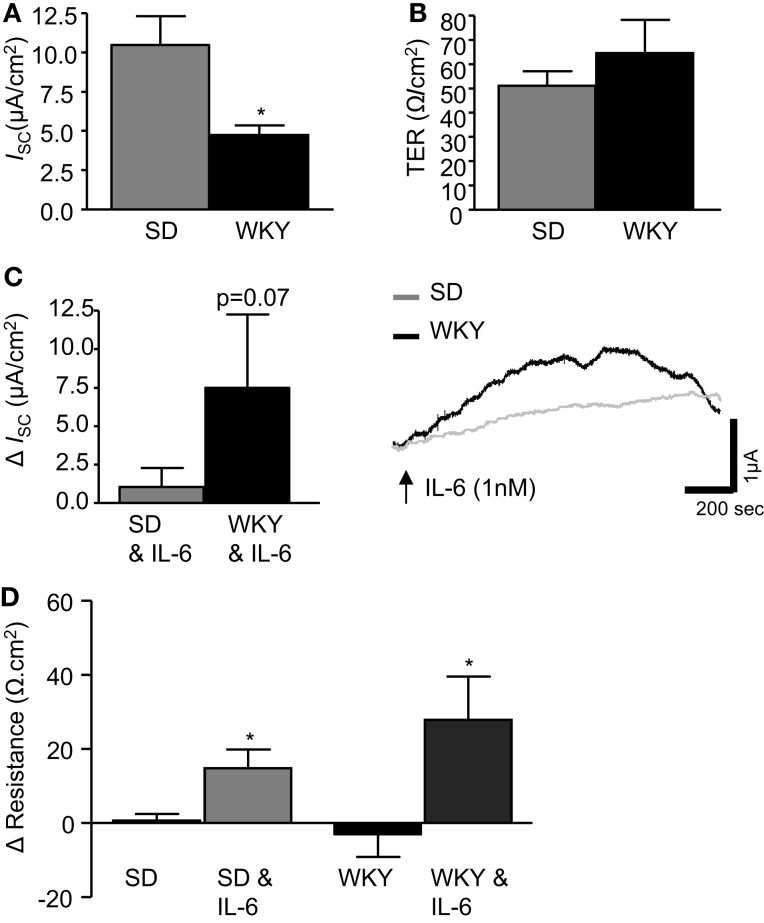
**IL-6 modulates transepithelial current and resistance**. **(A)** The histograms illustrates basal short circuit current (*I*_SC_, μA cm^2^, SD: *n* = 18, WKY: *n* = 9) and **(B)** transepithelial resistance (TER, Ω cm^2^, SD: *n* = 17, WKY: *n* = 8) in distal colon segments from naïve Sprague Dawley (SD) and Wistar Kyoto (WKY) rats. **(C)** The histograms and representative trace shows the effect of basolaterally applied IL-6 (1 nM, measured 10 min after application) on basal *I*_SC_ (SD: *n* = 23, WKY: *n* = 9). **(D)** This histogram illustrates the change (Δ) in tissue resistance over the course of the experiment (60–90 min, SD: *n* = 18, WKY: *n* = 6). Values = mean ± SEM.* Indicates *p* < 0.05.

Electrophysiological Ussing chamber studies were used to compare colonic transepithelial ion transfer and tissue resistance in the stress-sensitive WKY rats with the widely used SD comparator strain. Short circuit current (*I*_SC_) was measured and used as an indicator of net ionic movement across the tissue. In control colonic sections, not treated with IL-6, basal *I*_SC_ was found to be lower in WKY (*n* = 9) colonic sections as compared to SDs (*n* = 18, *p* < 0.05, Figure 1A). However, TER, an indicator of colonic permeability, was not different between SD (*n* = 17) and WKY (*n* = 8, *p* > 0.05, Figure 1B) tissues.

Previous studies in SD tissues determined that a peak increase in colonic *I*_SC_ was observed at ∼10 min during a 30 min application of IL-6 (1 nM) to the serosal reservoir (O’Malley et al., 2011b). Therefore, all measurements were taken at the 10 min timepoint in both SD and WKY colons. Replicating our previous findings (O’Malley et al., 2011b), IL-6 evoked a small increase in *I*_SC_ in SD controls (*n* = 23). Application of IL-6 to WKY tissue samples (*n* = 9) also induced a secretory current. However, the amplitude of the secretory response was larger in WKY tissues than SDs (*p* = 0.07, Figure 1C).

The change in TER was calculated by comparing a measurement of TER at the beginning and end of each experiment (60–90 min). In control tissue, not exposed to IL-6, no change was observed in TER in either SD or WKY rats (Figure 1D). However, the continued presence of IL-6 stimulated a significant increase in TER in both SD (12.6 ± 5.3 Ω cm^2^, *n* = 18) and WKY (27.8 ± 11.7 Ω cm^2^, *n* = 6, *p* > 0.05, Figure 1D) tissues. Two-way ANOVA analysis demonstrated a clear effect of the IL-6 treatment on tissue resistance [*F*_(1, 44)_ = 14.2, *p* < 0.001] but there were not any strain differences or interaction between the factors despite a trend toward a larger effect in the WKY tissue.

### *IL-6* potentiates veratridine-stimulated secretory currents in SD but not *WKY* rats

**Figure 2:**
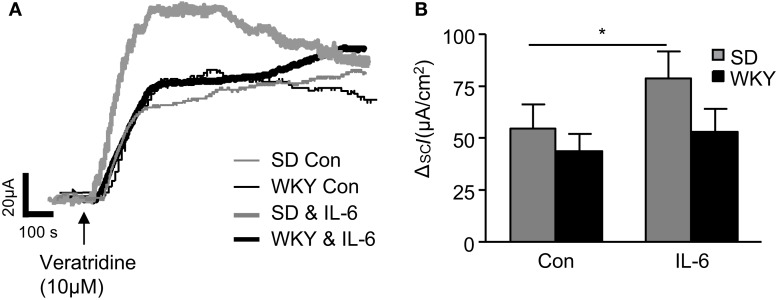
**Veratridine-induced currents in Sprague Dawley (SD) and Wistar Kyoto (WKY) colons**. **(A)** Representative traces illustrate the secretory current (*I*_SC_) induced by exposure to veratridine (10 μM) in non-stimulated (con) SD (thin gray line, *n* = 7) and WKY (thin black line, *n* = 9) colonic sections and IL-6-stimulated SD (thick gray line, *n* = 7) and WKY (thick black line, *n* = 9) colonic sections. **(B)** The histogram shows the pooled data. Values = mean ± SEM. * Indicates *p* < 0.05.

To assess differences in the sensitivity of neuronally mediated colonic secretion between SD and WKY rats, the sodium channel activator, veratridine (10 μM) was applied to the basolateral reservoir. In non-stimulated colon samples no differences were noted in the peak response to veratridine between SD (*n* = 19) and WKY (*n* = 10, *p* > 0.05, Figures 2A,B) rats. In paired experiments we found that exposure to IL-6 (1 nM, 30 min) potentiates the secretory effects of veratridine in SD tissues when compared to control non-stimulated samples (*n* = 15, *p* < 0.05), which is consistent with previous findings (O’Malley et al., 2011b). However, in WKY tissues, IL-6 exposure had no effect on veratridine-induced currents (*n* = 10, *p* > 0.05, Figures 2A,B).

### *IL-6* potentiates bethanechol-stimulated secretory currents

**Figure 3:**
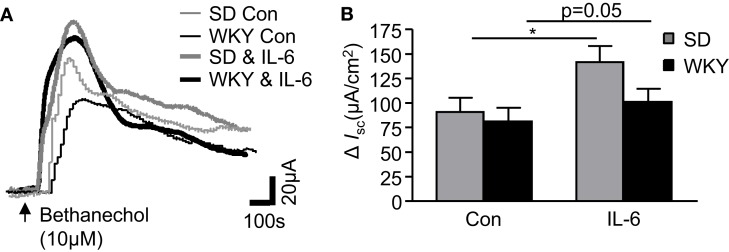
**Cholinergically driven currents in Sprague Dawley (SD) and Wistar Kyoto (WKY) rats are altered by IL-6 exposure**. **(A)** The representative traces show bethanechol-induced increases in short circuit current (*I*_SC_) in control (Con, thin gray line, *n* = 12) and IL-6-(1 nM) stimulated (IL-6, thick gray line, *n* = 12) SD colonic tissue. WKY traces are also included for control (thin black line, *n* = 9) and Il-6-stimulated (thick black line, *n* = 9) colonic tissues. **(B)** Histograms show the pooled data. Values = mean ± SEM. * Indicates *p* < 0.05.

To investigate strain differences in cholinergically mediated currents, the muscarinic receptor agonist, bethanechol (10 μM) was added to the basolateral chamber. The agonist evoked a rapid biphasic current in control SD tissues (*n* = 12). The peak response in WKY rats was comparable (*n* = 9, *p* > 0.05, Figures 3A,B). The modulatory effects of IL-6 on the bethanechol response were subsequently examined in both tissues. As we have previously demonstrated (O’Malley et al., 2011b), IL-6 potentiated the evoked bethanechol response in SD tissues (*n* = 12, *p* < 0.05). IL-6 also enhanced bethanechol-evoked secretion in WKY tissues (*n* = 9, *p* = 0.05, Figures 3A,B). Two-way ANOVA analysis demonstrated a significant effect of IL-6 treatment [*F*_(1, 38)_ = 5.4, *p* < 0.05], but no differences in strain or any interaction between the factors was identified.

Muscarinic acetylcholine receptors can be present on both epithelial cells and neurons in the gut. To determine which cell type excited by bethanechol were sensitive to the effects of IL-6, control experiments in SD tissues were carried out in the presence of the sodium channel blocker, tetrodotoxin. In paired experiments (*n* = 5 each), *I*_SC_ in IL-6-treated (98.8 ± 22.4 μA/cm^2^) and control (104 ± 29.7 μA/cm^2^) tissues following administration bethanechol in the presence of tetrodotoxin (100 nM, 15 min) were similar (*p* > 0.05). These data indicate that the potentiating effect of IL-6 on the bethanechol response appears to be mediated through neuronal activation.

### *IL-6* potentiates the anti-secretory phase of capsaicin-stimulated secretory currents in SD but not *WKY* rats

**Figure 4:**
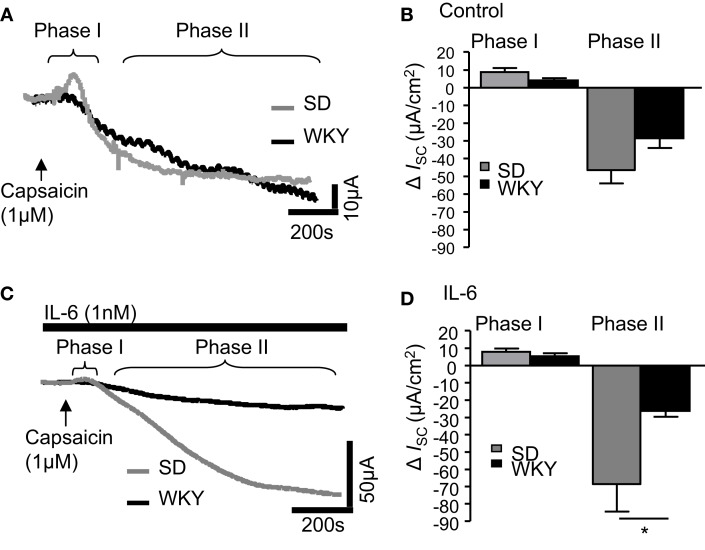
**IL-6 alters capsaicin-induced currents in Sprague Dawley (SD) and Wistar Kyoto (WKY) rat colons**. **(A)** The representative traces illustrate the secretory (phase I) and anti-secretory (phase II) responses to capsaicin (1 μM) in control SD (gray line, *n* = 10) and WKY (black line, *n* = 10) distal colons. **(B)** The histogram illustrates the pooled data for changes in current (*I*_SC_). **(C)** The traces are representative of capsaicin-evoked responses following exposure to IL-6 (1 nM) in SD (*n* = 11) and WKY (*n* = 7). **(D)** The pooled data are plotted in a histogram. Values = mean ± SEM. * indicates *p* < 0.05.

Finally, the sensory nerve stimulant capsaicin was examined in SD and WKY tissues. In control tissues, addition of capsaicin (1 μM) caused a rapid biphasic response as previously described (Yarrow et al., 1991). The early secretory phase (phase I) was comparable in both SD (*n* = 10) and WKY samples (*n* = 10, *p* > 0.05, Figures 4A,B). In phase II, where capsaicin evokes an anti-secretory current, *I*_SC_ values in non-stimulated SD and WKY tissues were also comparable (*p* > 0.05, Figures 4A,B). Pre-treatment with IL-6 (1 nM, 30 min) did not affect *I*_SC_ in phase I in either SD (*n* = 11) or WKY (*n* = 7) colons such that they remained comparable (*p* > 0.05, Figures 4C,D). However, IL-6 potentiated the capsaicin-evoked anti-secretory current in SD rats but not WKY rats such that a significant difference was apparent between the strains (*p* < 0.05, Figures 4C,D). Using two-way ANOVA, a difference in strain is approaching significance in the secretory phase [*F*_(1, 36)_ = 3.7, *p* = 0.06] with no effect of IL-6 and no interaction. In the anti-secretory phase, a strain difference is also apparent [*F*_(1, 31)_ = 7.7, *p* < 0.01] but there is no effect of the treatment itself and no interaction between the factors.

## Discussion

This series of electrophysiological studies builds on previous work from our group in which we demonstrated the capacity of IL-6 to directly stimulate a secretory current and decrease membrane permeability in colons from SD rats (O’Malley et al., 2011b). These studies have investigated the effects of IL-6 on colonic secretory and permeability parameters in WKY rats which exhibit several markers of GI dysfunction and have been used as an animal model of IBS. By comparing changes in colonic *I*_SC_ and TER between the strains, we have demonstrated that IL-6-induced changes in secretory activity and colonic permeability differ in WKY rats, thereby revealing a possible immune-mediated mechanism which could contribute to the dysfunctional bowel activity described in this rat (O’Malley et al., 2010a).

The WKY rat has been well characterized as a suitable pre-clinical model of IBS (Gunter et al., 2000; Gosselin et al., 2009; Gibney et al., 2010; O’Malley et al., 2010a). The GI dysfunction exhibited by the WKY rat includes an innate hypersensitivity to visceral pain stimuli such as that induced by colorectal distension (Gibney et al., 2010) and altered defecation patterns, particularly when exposed to stress (O’Malley et al., 2010a). Expression of the stress-related peptide, corticotropin-releasing factor (CRF) receptors are altered both in the colon (O’Malley et al., 2010b) and centrally (O’Malley et al., 2011d) in this strain. Given that amygdalar CRFR1 activation can contribute to visceral hypersensitivity in WKY rats (Johnson et al., 2012) these changes may have direct effects on the IBS-like symptom profile. Moreover, colonic toll-like receptor expression is also altered (McKernan et al., 2009) in this strain.

With regard to their colonic secretory parameters, WKY rats appear to display a pro-absorptive phenotype, which is thought to be reliant on decreased epithelial cholinergic sensitivity (Hyland et al., 2008). Under resting conditions we also observed this pro-absorptive phenotype as indicated by lower *I*_SC_ in the WKY rat as compared to SD. Interestingly, this relationship is reversed in the presence of IL-6, with a larger current being evoked from the WKY colonic tissues. The mechanisms underlying this secretory event are as yet unclear, however we have previously determined that submucosal neurons prepared from WKY colons display increased sensitivity to the neuroexcitatory effects of IL-6 (O’Malley et al., 2011a). As submucosal neurons have been attributed a primary role in regulating mucosal secretion and absorption, IL-6-mediated neural activation of colonic secretion may override the pro-absorptive phenotype regulated by epithelial cholinergic activity at rest (Hyland et al., 2008). This change in absorpto-secretory function would be consistent with the increase in mucus secretion and stress-induced fecal output evident in these animals (O’Malley et al., 2010a).

To further assess the importance of neurally evoked changes in secretion following application of IL-6 to the basolateral side of the tissue, pharmacological stimulators were applied as previously described (Julio-Pieper et al., 2010). Veratridine evokes neuronally mediated secretory currents by depolarizing intrinsic neurons via increased permeability through voltage-gated Na^+^ channels. This secretory response is caused by a net increase in Cl^−^ secretion (Sheldon et al., 1990). Under control conditions the veratridine-evoked responses were of similar amplitude in both SD and WKY colonic tissues and unlike SD tissue (O’Malley et al., 2011b), IL-6 had no effect on currents evoked in WKY tissue. Veratridine has been shown to stimulate the release of enteric neurotransmitters such as substance P, VIP, (Belai and Burnstock, 1988) and acetylcholine (Yau et al., 1986). However, further investigation will be required to determine the neurotransmitters underlying the IL-6-induced modulation of veratridine-stimulated ion secretion in SD rats. Evidently, this potentiating mechanism is not active in WKY rats.

The contribution of the cholinergic system to IL-6 secretion has been demonstrated in IBS patients (Dinan et al., 2008). Indeed, activation of secretomotor neurons may underlie neurogenic secretory diarrhea (Liebregts et al., 2007). In SD rats we provided evidence that IL-6 exposure potentiated currents induced by the muscarinic receptor agonist, bethanechol that were sensitive to the sodium channel blocker, tetrodotoxin. This effect was intact in WKY tissues as IL-6 similarly enhanced the bethanechol current.

Finally, currents evoked by activating transient receptor potential cation channels (TRPV1) were examined by exposing the tissue to capsaicin. Capsaicin stimulates visceral afferent neurons in the GI tract causing the subsequent release of nerve terminal neuropeptides which in turn, stimulate mucosal electrolyte transport and fluid secretion (Holzer et al., 1990; Vanner and MacNaughton, 1995), motility (Takaki and Nakayama, 1989), mucus secretion (Moore et al., 1996), and mucosal blood flow (Akiba et al., 1999) in addition to playing a protective role in maintaining mucosal integrity (Evangelista and Meli, 1989; Esplugues et al., 1990; Holzer et al., 1990). Indeed, TRPV1 is increased in inflammatory diseases of the GI tract (Yiangou et al., 2001) and in patients with rectal hypersensitivity (Chan et al., 2003). Application of capsaicin-evoked a large biphasic response in SD tissues which was comprised of an initial secretory phase followed by a larger more sustained anti-secretory phase consistent with previous studies (Yarrow et al., 1991). Interestingly, responses evoked by capsaicin in WKY tissues did not have such distinct phases. Rather than a small secretory response, there appeared to be a delay prior to the longer-lasting anti-secretory event, possibly indicating a balance between the two opposing mechanisms. Indeed, the differences between the strains came into sharper focus following addition of IL-6, which potentiated the anti-secretory phase in SD tissues only. Thus, in SD GI tissue, IL-6 exposure enhances the anti-secretory activity of afferent nerves whereas WKY rats have lost this regulatory response to IL-6 which could underlie the overall increased secretory activity in this strain. Moreover, the extrinsic, afferent innervation of the GI tract conveys information to the CNS that gives rise to the sensations of pain and discomfort. Thus, the insensitivity of WKY rats to IL-6-evoked potentiation of the capsaicin anti-secretory response may also be important in the increased sensitivity to visceral pain (Gibney et al., 2010).

Although the mechanisms of this effect require further elucidation, it is feasible that low-grade inflammation in the WKY rat may result in constant stimulation of capsaicin-sensitive nerve terminals causing neurotransmitter depletion or that IL-6 directly inhibits neurotransmitter release in these neurons. Alternatively, altered sensitivity to stress may contribute to these observations. CRF1 receptor antagonists alleviate visceral sensitivity in the WKY rat (Greenwood-Van Meerveld et al., 2005) but evidence exists for crosstalk between IL-6 and CRF (O’Malley et al., 2011c). Therefore, alterations in stress-induced expression of CRF receptors (O’Malley et al., 2010b; O’Malley et al., 2011d), may be linked to the increase in IL-6 sensitivity observed in the WKY rats.

Tissue resistance was also measured in these animals as a marker of membrane permeability and was found to be similar between strains. Interestingly, IL-6 stimulated an increase in TER in both strains. Changes in permeability can occur as a result of alterations in the expression of tight junctions (Chen et al., 2010; Martinez et al., 2012), dysbiosis of microbiota (Fukuda et al., 2011) or through the increased presence of pro-inflammatory cytokines (Arrieta et al., 2006). Furthermore, stress can contribute to changes in permeability as has previously been demonstrated in WKY rats (Saunders et al., 2002). Over the 90 min duration of these recordings, one possibility might be an increase in mucus secretion evoked by IL-6, which could influence membrane permeability. Indeed, we have previously observed increased expression of goblet cell number in the WKY strain (O’Malley et al., 2010a). We demonstrated that acute application of IL-6 increases TER, appearing to help maintain the integrity of the epithelial cell layer in both SD and WKY rats. This is consistent with one previous study (Wang et al., 2007). On the other hand, chronic exposure to elevated IL-6 levels may result in increased gut permeability (Hiribarren et al., 1993; Natale et al., 2003). As mucosal levels of the pro-inflammatory cytokine, IL-6 are elevated in WKY colons (O’Malley et al., 2011a), one might have expected that continuous exposure to IL-6 would have resulted in increased colonic permeability in this strain. However, TER at rest is comparable between SD and WKY rats. This may indicate that there are increased numbers of IL-6-containing cells in the mucosa of WKY rats but not necessarily increased levels of IL-6 secretion.

At a functional level, these studies have demonstrated that IL-6-evoked secretion is enhanced in WKY colons and this is likely to be due to increased sensitivity of submucosal neurons to the pro-inflammatory cytokine. We have provided evidence that inhibition of the potentiating effect of IL-6 on capsaicin-evoked anti-secretory currents is a likely contributor to the changes in colonic secretion. These data further demonstrate the neuromodulatory effects of IL-6 in colonic function and provide mechanistic evidence of how elevations in systemic IL-6 in IBS patients could be a contributory factor in the pathophysiology of the disorder.

## Contribution of Each Author

Dervla O’Malley: study concept and design; acquisition of data; analysis and interpretation of data; drafting of the manuscript; critical revision of the manuscript for important intellectual content; statistical analysis. Timothy G. Dinan: critical revision of the manuscript for important intellectual content; study supervision. John F. Cryan: critical revision of the manuscript for important intellectual content; study supervision.

## Conflict of Interest Statement

The authors declare that the research was conducted in the absence of any commercial or financial relationships that could be construed as a potential conflict of interest.

## References

[B1] Akiba Y. Guth P. H. Engel E. Nastaskin I. Kaunitz J. D. (1999). Acid-sensing pathways of rat duodenum. Am. J. Physiol. 277, G268–G274 10444439 10.1152/ajpgi.1999.277.2.G268

[B2] Al-Sadi R. Boivin M. Ma T. (2009). Mechanism of cytokine modulation of epithelial tight junction barrier. Front. Biosci. 14, 2765–2778 10.2741/3413 19273235 PMC3724223

[B3] Arrieta M. C. Bistritz L. Meddings J. B. (2006). Alterations in intestinal permeability. Gut 55, 1512–1520 10.1136/gut.2005.085373 16966705 PMC1856434

[B4] Belai A. Burnstock G. (1988). Release of calcitonin gene-related peptide from rat enteric nerves is Ca2+-dependent but is not induced by K+ depolarization. Regul. Pept. 23, 227–235 10.1016/0167-0115(88)90030-4 2466307

[B5] Camilleri M. Madsen K. Spiller R. Van Meerveld B. G. Verne G. N. (2012). Intestinal barrier function in health and gastrointestinal disease. Neurogastroenterol. Motil. 24, 503–512 10.1111/j.1365-2982.2012.01921.x 22583600 PMC5595063

[B6] Chadwick V. S. Chen W. Shu D. Paulus B. Bethwaite P. Tie A. (2002). Activation of the mucosal immune system in irritable bowel syndrome. Gastroenterology 122, 1778–1783 10.1053/gast.2002.33579 12055584

[B7] Chan C. L. Facer P. Davis J. B. Smith G. D. Egerton J. Bountra C. (2003). Sensory fibres expressing capsaicin receptor TRPV1 in patients with rectal hypersensitivity and faecal urgency. Lancet 361, 385–391 10.1016/S0140-6736(03)12280-5 12573376

[B8] Chen H. Q. Yang J. Zhang M. Zhou Y. K. Shen T. Y. Chu Z. X. (2010). Lactobacillus plantarum ameliorates colonic epithelial barrier dysfunction by modulating the apical junctional complex and PepT1 in IL-10 knockout mice. Am. J. Physiol. Gastrointest. Liver Physiol. 299, G1287–G1297 10.1152/ajpgi.00364.2009 20884889

[B9] Clarke G. Quigley E. M. Cryan J. F. Dinan T. G. (2009). Irritable bowel syndrome: towards biomarker identification. Trends. Mol. Med. 15, 478–489 10.1016/j.molmed.2009.08.001 19811951

[B10] Dinan T. G. Clarke G. Quigley E. M. Scott L. V. Shanahan F. Cryan J. (2008). Enhanced cholinergic-mediated increase in the pro-inflammatory cytokine IL-6 in irritable bowel syndrome: role of muscarinic receptors. Am. J. Gastroenterol. 103, 2570–2576 10.1111/j.1572-0241.2008.01871.x 18785949

[B11] Dinan T. G. Quigley E. M. Ahmed S. M. Scully P. O’Brien S. O’Mahony L. (2006). Hypothalamic-pituitary-gut axis dysregulation in irritable bowel syndrome: plasma cytokines as a potential biomarker? Gastroenterology 130, 304–311 10.1053/j.gastro.2005.11.033 16472586

[B12] Esplugues J. V. Ramos E. G. Gil L. Esplugues J. (1990). Influence of capsaicin-sensitive afferent neurones on the acid secretory responses of the rat stomach in vivo. Br. J. Pharmacol. 100, 491–496 10.1111/j.1476-5381.1990.tb15835.x 2202478 PMC1917796

[B13] Evangelista S. Meli A. (1989). Influence of capsaicin-sensitive fibres on experimentally-induced colitis in rats. J. Pharm. Pharmacol. 41, 574–575 10.1111/j.2042-7158.1989.tb06532.x 2571707

[B14] Fitzgerald P. Cassidy Eugene M. Clarke G. Scully P. Barry S. Quigley Eamonn M. M. (2008). Tryptophan catabolism in females with irritable bowel syndrome: relationship to interferon-gamma, severity of symptoms and psychiatric co-morbidity. Neurogastroenterol. Motil. 20, 1291–1297 10.1111/j.1365-2982.2008.01195.x 18823288

[B15] Fukuda S. Toh H. Hase K. Oshima K. Nakanishi Y. Yoshimura K. (2011). Bifidobacteria can protect from enteropathogenic infection through production of acetate. Nature 469, 543–547 10.1038/nature09646 21270894

[B16] Gibney S. M. Gosselin R. D. Dinan T. G. Cryan J. F. (2010). Colorectal distension-induced prefrontal cortex activation in the Wistar-Kyoto rat: implications for irritable bowel syndrome. Neuroscience 165, 675–683 10.1016/j.neuroscience.2009.08.076 19765638

[B17] Gosselin R. D. Gibney S. O’Malley D. Dinan T. G. Cryan J. F. (2009). Region specific decrease in glial fibrillary acidic protein immunoreactivity in the brain of a rat model of depression. Neuroscience 159, 915–925 10.1016/j.neuroscience.2008.10.018 19000745

[B18] Greenwood-Van Meerveld B. Johnson A. C. Cochrane S. Schulkin J. Myers D. A. (2005). Corticotropin-releasing factor 1 receptor-mediated mechanisms inhibit colonic hypersensitivity in rats. Neurogastroenterol. Motil. 17, 415–422 10.1111/j.1365-2982.2005.00648.x 15916629

[B19] Gunter W. D. Shepard J. D. Foreman R. D. Myers D. A. Greenwood-Van Meerveld B. (2000). Evidence for visceral hypersensitivity in high-anxiety rats. Physiol. Behav. 69, 379–382 10.1016/S0031-9384(99)00254-1 10869605

[B20] Hiribarren A. Heyman M. L’helgouac’h A. Desjeux J. F. (1993). Effect of cytokines on the epithelial function of the human colon carcinoma cell line HT29 cl 19A. Gut 34, 616–620 10.1136/gut.34.5.616 8504961 PMC1374177

[B21] Holzer P. Pabst M. A. Lippe I. T. Peskar B. M. Peskar B. A. Livingston E. H. (1990). Afferent nerve-mediated protection against deep mucosal damage in the rat stomach. Gastroenterology 98, 838–848 10.1016/0016-5085(90)90005-L 2311873

[B22] Hurst S. M. Stanisz A. M. Sharkey K. A. Collins S. M. (1993). Interleukin 1 beta-induced increase in substance P in rat myenteric plexus. Gastroenterology 105, 1754–1760 7504644 10.1016/0016-5085(93)91073-q

[B23] Hyland N. P. G. S. Gosselin R. D. Lee K. Dinan T. G. Cryan J. F. (2008). Assessment of colonic secretory function and faecal output in viscerally hypersensitive Wistar Kyoto rats. Gastroenterology, abstr. A277. 10.1016/S0016-5085(08)61290-1

[B24] Johnson A. C. Tran L. Schulkin J. Greenwood-Van Meerveld B. (2012). Importance of stress receptor-mediated mechanisms in the amygdala on visceral pain perception in an intrinsically anxious rat. Neurogastroenterol. Motil. 24, 479–486 10.1111/j.1365-2982.2012.01899.x 22364507 PMC3461498

[B25] Julio-Pieper M. Hyland N. P. Bravo J. A. Dinan T. G. Cryan J. F. (2010). A novel role for the metabotropic glutamate receptor-7: modulation of faecal water content and colonic electrolyte transport in the mouse. Br. J. Pharmacol. 160, 367–375 10.1111/j.1476-5381.2010.00713.x 20423346 PMC2874858

[B26] Kelles A. Janssens J. Tack J. (2000). IL-1beta and IL-6 excite neurones and suppress cholinergic neurotransmission in the myenteric plexus of the guinea pig. Neurogastroenterol. Motil. 12, 531–538 10.1046/j.1365-2982.2000.00228.x 11123708

[B27] Liebregts T. Adam B. Bredack C. Roth A. Heinzel S. Lester S. (2007). Immune activation in patients with irritable bowel syndrome. Gastroenterology 132, 913–920 10.1053/j.gastro.2007.01.046 17383420

[B28] Lydiard R. B. Fossey M. D. Marsh W. Ballenger J. C. (1993). Prevalence of psychiatric disorders in patients with irritable bowel syndrome. Psychosomatics 34, 229–234 10.1016/S0033-3182(93)71884-8 8493304

[B29] Macsharry J. O’Mahony L. Fanning A. Bairead E. Sherlock G. Tiesman J. (2008). Mucosal cytokine imbalance in irritable bowel syndrome. Scand. J. Gastroenterol. 43, 1467–1476 10.1080/00365520802276127 18752146

[B30] Martinez C. Vicario M. Ramos L. Lobo B. Mosquera J. L. Alonso C. (2012). The jejunum of diarrhea-predominant irritable bowel syndrome shows molecular alterations in the tight junction signaling pathway that are associated with mucosal pathobiology and clinical manifestations. Am. J. Gastroenterol. 107, 736–746 10.1038/ajg.2011.472 22415197

[B31] McKernan D. P. Gaszner G. Quigley E. M. Cryan J. F. Dinan T. G. (2011). Altered peripheral toll-like receptor responses in the irritable bowel syndrome. Aliment. Pharmacol. Ther. 33, 1045–1052 10.1111/j.1365-2036.2011.04624.x 21453321

[B32] McKernan D. P. Nolan A. Brint E. K. O’Mahony S. M. Hyland N. P. Cryan J. F. (2009). Toll-like receptor mRNA expression is selectively increased in the colonic mucosa of two animal models relevant to irritable bowel syndrome. PLoS ONE 4, e8226 10.1371/journal.pone.0008226 20011045 PMC2785428

[B33] Moore B. A. Sharkey K. A. Mantle M. (1996). Role of 5-HT in cholera toxin-induced mucin secretion in the rat small intestine. Am. J. Physiol. 270, G1001–G1009 8764208 10.1152/ajpgi.1996.270.6.G1001

[B34] Natale L. Piepoli A. L. De Salvia M. A. De Salvatore G. Mitolo C. I. Marzullo A. (2003). Interleukins 1 beta and 6 induce functional alteration of rat colonic motility: an in vitro study. Eur. J. Clin. Invest. 33, 704–712 10.1046/j.1365-2362.2003.01200.x 12864781

[B35] O’Malley D. Dinan T. G. Cryan J. F. (2011a). Altered expression and secretion of colonic interleukin-6 in a stress-sensitive animal model of brain-gut axis dysfunction. J. Neuroimmunol. 235, 48–55 10.1016/j.jneuroim.2011.04.003 21565410

[B36] O’Malley D. Liston M. Hyland N. P. Dinan T. G. Cryan J. F. (2011b). Colonic soluble mediators from the maternal separation model of irritable bowel syndrome activate submucosal neurons via an interleukin-6-dependent mechanism. Am. J. Physiol. Gastrointest. Liver Physiol. 300, G241–G252 10.1152/ajpgi.00385.2010 21109592

[B37] O’Malley D. Dinan T. G. Cryan T. F. (2011d). Neonatal maternal separation in the rat impacts on the stress-responsivity of central corticotropin-releasing factor receptors in adulthood. Psychopharmacology (Berl.) 214, 221–229 10.1007/s00213-010-1885-9 20499051

[B38] O’Malley D. Quigley E. M. Dinan T. G. Cryan J. F. (2011c). Do interactions between stress and immune responses lead to symptom exacerbations in irritable bowel syndrome? Brain Behav. Immun. 25, 1333–1341 10.1016/j.bbi.2011.04.009 21536124

[B39] O’Malley D. Julio-Pieper M. Gibney S. M. Dinan T. G. Cryan J. F. (2010a). Distinct alterations in colonic morphology and physiology in two rat models of enhanced stress-induced anxiety and depression-like behaviour. Stress 13, 114–122 10.3109/10253890903067418 20214436

[B40] O’Malley D. Julio-Pieper M. Gibney S. M. Gosselin R. D. Dinan T. G. Cryan J. F. (2010b). Differential stress-induced alterations of colonic corticotropin-releasing factor receptors in the Wistar Kyoto rat. Neurogastroenterol. Motil. 22, 301–311 10.1111/j.1365-2982.2009.01412.x 19807869

[B41] Quigley E. M. (2006). Changing face of irritable bowel syndrome. World J. Gastroenterol. 12, 1–5 16440408 10.3748/wjg.v12.i1.1 PMC4077493

[B42] Ruhl A. Franzke S. Collins S. M. Stremmel W. (2001). Interleukin-6 expression and regulation in rat enteric glial cells. Am. J. Physiol. Gastrointest. Liver Physiol. 280, G1163–G1171 11352809 10.1152/ajpgi.2001.280.6.G1163

[B43] Ruhl A. Hurst S. Collins S. M. (1994). Synergism between interleukins 1 beta and 6 on noradrenergic nerves in rat myenteric plexus. Gastroenterology 107, 993–1001 7926489 10.1016/0016-5085(94)90223-2

[B44] Saunders P. R. Maillot C. Million M. Tache Y. (2002). Peripheral corticotropin-releasing factor induces diarrhea in rats: role of CRF1 receptor in fecal watery excretion. Eur. J. Pharmacol. 435, 231–235 10.1016/S0014-2999(01)01574-6 11821031

[B45] Scully P. Mckernan D. P. Keohane J. Groeger D. Shanahan F. Dinan T. G. (2010). Plasma cytokine profiles in females with irritable bowel syndrome and extra-intestinal co-morbidity. Am. J. Gastroenterol 105, 2235–2243 10.1038/ajg.2010.159 20407431

[B46] Sheldon R. J. Malarchik M. E. Burks T. F. Porreca F. (1990). Effects of nerve stimulation on ion transport in mouse jejunum: responses to Veratrum alkaloids. J. Pharmacol. Exp. Ther. 252, 636–642 2313592

[B47] Spiller R. C. (2004). Irritable bowel syndrome. Br. Med. Bull. 72, 15–29 10.1093/bmb/ldh039 15767561

[B48] Takaki M. Nakayama S. (1989). Effects of capsaicin on myenteric neurons of the guinea pig ileum. Neurosci. Lett. 105, 125–130 10.1016/0304-3940(89)90023-2 2485875

[B49] Vanner S. MacNaughton W. K. (1995). Capsaicin-sensitive afferent nerves activate submucosal secretomotor neurons in guinea pig ileum. Am. J. Physiol. 269, G203–G209 7653559 10.1152/ajpgi.1995.269.2.G203

[B50] Wang L. Srinivasan S. Theiss A. L. Merlin D. Sitaraman S. V. (2007). Interleukin-6 induces keratin expression in intestinal epithelial cells: potential role of keratin-8 in interleukin-6-induced barrier function alterations. J. Biol. Chem. 282, 8219–8227 10.1074/jbc.M703408200 17213200

[B51] Xia Y. Hu H. Z. Liu S. Ren J. Zafirov D. H. Wood J. D. (1999). IL-1beta and IL-6 excite neurons and suppress nicotinic and noradrenergic neurotransmission in guinea pig enteric nervous system. J. Clin. Invest. 103, 1309–1316 10.1172/JCI5823 10225974 PMC408357

[B52] Yarrow S. Ferrar J. A. Cox H. M. (1991). The effects of capsaicin upon electrogenic ion transport in rat descending colon. Naunyn Schmiedebergs Arch. Pharmacol. 344, 557–563 10.1007/BF00170652 1725806

[B53] Yau W. M. Dorsett J. A. Youther M. L. (1986). Calcium-dependent stimulation of acetylcholine release by substance P and vasoactive intestinal polypeptide. Eur. J. Pharmacol. 120, 241–243 10.1016/0014-2999(86)90547-9 2419150

[B54] Yiangou Y. Facer P. Dyer N. H. Chan C. L. Knowles C. Williams N. S. (2001). Vanilloid receptor 1 immunoreactivity in inflamed human bowel. Lancet 357, 1338–1339 10.1016/S0140-6736(00)04503-7 11343743

